# Promoting patients’ rights through hospital accreditation

**DOI:** 10.1186/s13584-020-00421-1

**Published:** 2020-11-30

**Authors:** Carsten Engel

**Affiliations:** 1 IKAS, Danish Institute for Quality and Accreditation in Healthcare, Aarhus N, Denmark; 2 ISQua International Academy of Quality and Safety (IAQS), Aarhus N, Denmark

**Keywords:** Accreditation, Patient’s rights, Health quality, Regulation

## Abstract

Legislation and accreditation standards both address patients’ rights. The two approaches differ in important ways; they should not be seen as competing but as complementing efforts. Laws define minimum standards, whereas accreditation standards describe optimal performance; laws focus on the rights, whereas accreditation standards also point out ways in which hospitals may act to deliver these rights, which both serves to help hospitals implementing the rights and to standardize the measures taken across hospitals. A recent Israeli study underpins this view, but also highlights that international accreditation standards and national legislation may differ, when it comes to the definition of the actual rights.

## Main text

Accreditation is widely used to promote and improve quality in hospitals and other healthcare provider organizations; often it is carried out under the umbrella of ISQua’s International Accreditation Programme [1]. Traditionally, accreditation has been a voluntary process, but in many countries it has become mandated, either de facto, i.e. as a prerequisite for receiving funding from public funds or insurance companies, or de jure, as a statutory requirement. The latter has been the case for hospitals in Israel since 2012.

Suppose that a country already has well-developed regulatory requirements for the provision of healthcare, what then is the value of adding accreditation on top of this – and what frictions should we expect? Obviously, accreditation could serve as an outsourced form of inspection, i.e. as an external and independent evaluation of compliance with the regulatory requirements; accreditation bodies have the expertise to perform this type of evaluation. But could accreditation provide more value than just an assessment?

In their paper in the Israel Journal of Health Policy Research [2], Sperling and Pikkel present an analysis that provides important contributions to the answer to this question. The authors have examined the scope, contents, and definitions of patients’ rights in the Joint Commission International (JCI) Standards and compared them to patients’ rights as they are addressed and protected in national Israeli legislation. As expected, they found a considerable overlap; after all, patients’ rights is a universally acknowledged concept, protected by international law. Nevertheless, they identified some important differences in content, most strikingly the almost complete absence of the term “family” from Israeli legislation, contrasted to the strong emphasis of the rights of both patients and families in the JCI Standards.

A potential source of friction is introduced, when an international set of standards is incorporated into the national legal framework, introducing new requirements and raising questions of whether failure to act as required by the accreditation standards could lead to legal action against a hospital. This friction could be avoided by developing a national accreditation programme that can easily be tailored to correspond to local legislation. On the other hand, comparing national understanding of concepts such as patients’ rights to international standards may provide inspiration and highlight possible blind spots. And accreditation by international standards may be preferred from numerous other considerations, such as the costs associated with developing a national programme or a desire to demonstrate compliance with international standards.

When considering the potential friction, one should keep in mind that while state licensing aims at ensuring *minimal* service standards are met by each organization, as defined by the legislator, the accreditation process aims at ensuring *optimal* standards of service. The prudent policy maker will wish both to declare a minimum level, below which reprisals and/or enforced corrective measures will be incurred, and to declare expectations for where healthcare is heading. Accreditation standards offer an opportunity to express these expectations, while acknowledging that it is not anticipated that every expectation is met from the very moment it has been stated. Accreditation does not only call for compliance, it also calls for movement, improvement. Failure to comply fully with accreditation standards should not per se constitute a basis for litigation or be viewed as negligence; failure to improve might.

However, in my view, perhaps the most interesting difference between legislation and accreditation is the way in which the two present patients’ rights to those who must implement these rights. Sterling and Pikkel point out that the law details what the rights are; it sets the general foundations for the rights that are at stake. Such a foundation is clearly needed. We need to be clear about what we are aiming for, and we need a yardstick by which to measure whether a patient’s rights were respected or violated on a specific occasion. Merely studying this foundation, however, may leave the hospitals in doubt about what they actually must do, which in turn may lead to both delayed implementation and unwanted variations in actions taken.

The accreditation standards translate the rights into actions. They aim at ensuring that hospitals perform certain activities in line with existing standards by setting expectations for specific structures and processes that should be in place in the hospital. This both facilitates implementation and reduces variation, while standards can be designed in ways that still leave sufficient room for necessary local adaptations – and for innovative approaches. Furthermore, standards integrate content that in the Israeli case is spread across five different laws and present it in a language that is targeted at healthcare providers, not the legal professions.

Many hospitals are exposed to two sets of potentially uncoordinated regulations, law and accreditation standards; studies, where the two are compared in such detail as Sperling and Pikkel have done, are very useful. This particular case highlights specific sources for friction, and also adds to our general understanding of their interaction.

## Conclusions

The paper by Sperling and Pikkel helps us appreciate how accreditation standards may support and complement law. Accreditation provides policy makers with an opportunity to express expectations for an optimum, in addition to requirements for a minimum, in other words to draw a roadmap for the continuous journey of improvement. Accreditation can be used to promote aspirations that are not suitable for, or not yet ripe for, formulation as statutory rights or duties.

Accreditation, moreover, provides a way to translate the language of the law into an actionable language as outlined above, thereby demonstrating ways in which the intentions of the legislators could or should be realized. The dialogue with peer reviewers during an accreditation survey reinforces the learning effect.

Another group of authors has called this translational role “an unrecognized power of accreditation” [3]. I share their opinion that we will only achieve the full benefit of the accreditation process if we understand how to use it as a knowledge-to-action intervention to bring about meaningful and sustained change.

## Data Availability

Not relevant.

## Abbreviations

ISQua: International Society for Quality in Health Care
JCI: Joint Commission International
